# Socio-Demographic Factors Associated with Alcohol and Tobacco Use Among Virally Suppressed People Living with HIV in the Eastern Cape, South Africa

**DOI:** 10.3390/tropicalmed11080230

**Published:** 2026-08-18

**Authors:** Zanele Bennedict Nomatshila, Guillermo Alfredo Pulido Estrada, Sibusiso Cyprian Nomatshila, Teke Ruffin Apalata

**Affiliations:** 1 School of Public Health, Faculty of Medicine and Health Sciences, Walter Sisulu University, Mthatha 5117, South Africa; gpulidoestrada@wsu.ac.za (G.A.P.E.); snomatshila@wsu.ac.za (S.C.N.); 2 Department of Social Work, Faculty of Law, Humanities and Social Science, Walter Sisulu University, Mthatha 5117, South Africa; 3 Faculty of Health Sciences, University of Stirling, Stirling FK9 4LA, UK; tapalata@wsu.ac.za; 4 Biostatistics and Analytics Training Services, Faculty of Medicine and Health Sciences, Walter Sisulu University, Mthatha 5117, South Africa; 5 Society and Health Research Institute, Faculty of Medicine and Health Sciences, Walter Sisulu University, Mthatha 5117, South Africa; 6 School of Pathology, Faculty of Medicine and Health Sciences, Walter Sisulu University, Mthatha 5117, South Africa

**Keywords:** viral load suppression, alcohol, quality of life, tobacco smoking, HIV, antiretroviral therapy, health behaviour

## Abstract

Background: Alcohol and tobacco use remain important public health concerns among people living with HIV (PLWH), even after achieving viral load suppression. This study examined the socio-demographic factors associated with alcohol and tobacco use among virally suppressed PLWH in the Eastern Cape Province, South Africa. Methods: A cross-sectional study was conducted among 244 adults receiving antiretroviral therapy with suppressed viral loads at public healthcare facilities in the Eastern Cape Province. Data were collected using the Alcohol Use Disorders Identification Test (AUDIT) and the WHO STEPwise questionnaire. Alcohol use was classified according to the AUDIT, and participants were further categorised into low-risk (AUDIT score < 8) and risky drinking (AUDIT score ≥ 8) for regression analyses. Associations between socio-demographic characteristics and alcohol use were initially examined using cross-tabulations and exact tests where appropriate. Univariable and multivariable binary logistic regression analyses were subsequently performed to identify factors independently associated with risky alcohol use and smoking. Adjusted odds ratios (AORs) with 95% confidence intervals (CIs) were reported, with statistical significance set at *p* < 0.05. Results: Of the 244 participants, 57.0% were female, and 61.5% were employed. Based on the total AUDIT score, 60.2% of participants were classified as risky drinkers, while 44.3% were current smokers. In the multivariable analysis, employment was independently associated with risky alcohol use (AOR = 2.03, 95% CI: 1.11–3.73) and current smoking (AOR = 3.75, 95% CI: 1.98–7.08). Compared with single participants, married (AOR = 2.61, 95% CI: 1.25–5.45), separated (AOR = 5.19, 95% CI: 1.98–13.60), and divorced participants (AOR = 3.32, 95% CI: 1.05–10.49) had higher odds of risky alcohol use. Conclusion: Alcohol use and tobacco smoking remain common among virally suppressed PLWH in the Eastern Cape Province. Although viral load suppression is an important treatment outcome, it does not fully reflect broader health behaviours that may compromise long-term health. Integrating routine screening for alcohol and tobacco use, together with targeted behavioural interventions and harm reduction strategies, into comprehensive HIV care may improve long-term health outcomes.

## 1. Introduction

The introduction of antiretroviral treatment (ART) for PLWH has led to improved health outcomes, longer life expectancy, and better quality of life [1]. Among the 39.9 million individuals reported to be HIV positive globally in 2023, viral load suppression was achieved among 77%, which extended life expectancy for those on ART [2]. This was linked to adjustments in health behaviours such as alcohol consumption and/or tobacco use, which are considered to have a significant influence on the overall health and well-being of PLWH [3]. The evidence concerning the effects of alcohol on HIV medication is characterised by both diversity and complexity, presenting mixed findings and nuanced perspectives. Alcohol and/or tobacco use present considerable health risks for PLWH. Chronic and excessive alcohol use can impair immune function, thereby complicating the management of HIV. Furthermore, alcohol may interact adversely with specific antiretroviral medications, potentially diminishing treatment adherence and overall effectiveness [3]. Myers et al. [4] established a statistical association between alcohol consumption and reduced efficacy in viral load (VL) suppression among PLWH. This finding emphasises a critical public health issue. However, it is essential to acknowledge that the association between alcohol consumption and VL suppression may be modulated by various factors, including adherence to ART and individual drinking patterns. Moreover, the specific biological mechanisms underlying this association remain under investigation.

Achieving undetectable viral loads is essential for minimising the transmissibility of HIV. This objective can be more effectively attained through various lifestyle modifications, particularly through the reduction of alcohol consumption and the cessation of smoking. These adjustments not only contribute to improved overall health but also play a significant role in enhancing adherence to antiretroviral therapy, ultimately supporting viral suppression [5].

Beyond achieving viral load suppression, additional behaviours, like alcohol and tobacco use, represent important barriers to the overall health and well-being of PLWH [6]. There is evidence suggesting that alcohol use may have a greater impact on the mortality and morbidity of PLWH than on the general population, suggesting that viral load suppression may be inadequate but requires support through healthy behavioural choices [3]. South Africa is reported to have one of the highest levels of alcohol consumption per capita in the world, making substance abuse a serious public health concern [7]. Furthermore, 7% of women in South Africa report hazardous drinking, which is defined as alcohol use that raises the chance of negative effects [7]. Egbe et al. emphasise that alcohol use impairs the health of those living with HIV [8]. Unhealthy alcohol intake can interfere negatively with the immune system, impairing ART adherence and worsening health risks such as cardiovascular disease, non-AIDS-defining diseases, and mental health difficulties [9]. Velloza et al. also indicate that PLWH who use substances are more likely to be ART non-adherent than those who do not disclose the use of substances, thus compromising their body’s ability to respond to infections [10].

Tobacco smoking has severe health consequences, affecting multiple organ systems and increasing the risk of chronic diseases [11]. These health hazards are substantial for all smokers, but especially for those living with HIV, who smoke at about double the rate of the general population [12]. It is reported that PLWH already have a compromised immune system due to their HIV status, but the use of tobacco products exacerbates this, increasing morbidity and mortality rates [7]. Smoking PLWH are more likely to respond poorly to HIV therapy and have a shorter lifetime than those who do not smoke [13].

The consumption of alcohol and tobacco is prevalent among PLWH and is influenced by a range of socio-demographic factors, including age, gender, race, income level, and mental health status [14]. Furthermore, the intersectionality of race and socioeconomic background complicates our understanding of drug use behaviours among PLWH. Higher levels of educational attainment, such as having some postsecondary education, along with employment and income, are associated with lower odds of alcohol use [15]. There is a lack of research on alcohol and tobacco use among patients in South Africa who have achieved viral load suppression. Additionally, there are still gaps in how to manage the quality of life for people living with HIV after they reach this goal, particularly related to tobacco and alcohol use.

Alcohol and tobacco use are influenced by multiple interrelated socio-demographic factors, including age, gender, educational attainment, employment, and marital status. Because these characteristics may confound one another, examining each factor independently may not accurately identify those associated with substance use. Therefore, in addition to descriptive and bivariate analyses, multivariable logistic regression was used to determine the independent association between socio-demographic characteristics and alcohol and tobacco use while adjusting for potential confounding variables.

On this basis, the aim of this study was to examine socio-demographic associations of alcohol and tobacco use among PLWH, who have achieved viral load suppression in the Eastern Cape in South Africa.

## 2. Materials and Methods

### 2.1. Study Design

This study employed a cross-sectional design to enrol PLWH who had achieved viral load suppression in the selected study site.

### 2.2. Study Setting

This study was conducted in selected health facilities in the Eastern Cape Province of South Africa. The Eastern Cape province is one of the most rural provinces, with a population of more than 7.2 million [16]. The majority (85%) of the province’s population is African, with 65% aged 15–65 years [16]. Only 10% of the population above 20 years has higher education, and the employment rate is recorded at 42% as of 2022 [16].

### 2.3. Study Population

The study population comprised PLWH aged 18 years and above, who are receiving ART and had achieved viral load suppression (<200 copies of HIV per millilitre of blood) in the Eastern Cape province, South Africa. Any participant whose viral load was not suppressed was excluded from the study.

### 2.4. Sampling Procedure

Cluster sampling was used to select health facilities in the OR Tambo, Alfred Nzo, Joe Gqabi, and Chris Hani districts of the Eastern Cape province. A total of 11 randomly selected clinics were included, representing both community health centres (*n* = 3) and clinics (*n* = 8). Participants were selected by a team of researchers using simple random sampling from the database of PLWH receiving ART who had achieved viral load suppression (participants with a laboratory test of less than 200 copies/mL). Eligible participants in this current study should have been part of the 1300 PLWH who participated in the Research Capacity Development Initiative (RCDI) study (MRC-RFACC 01-2014) conducted by Walter Sisulu University and should have consented to participate in subsequent HIV-related studies. The Authors in the current study were co-authors in the RCDI study. Only participants aged 18 years with VL who gave written informed consent to participate in the current study were considered. Those with cognitive impairments or who were unable to provide consent were excluded from the study.

### 2.5. Sample Size Calculation

The Cochran formula [17] was used to calculate the appropriate sample size for this study The initial sample size target was 267 PLWH (also 20% proportionate size of SAMRC-RCDI study) who had achieved viral load suppression. To account for sampling variability and potential biases, a 10% adjustment was applied, resulting in a final sample size ranging from 294 (maximum) to 240 (minimum).

### 2.6. Data Collection

Trained research assistants collected data using standardised, validated researcher-administered questionnaires to record sociodemographic characteristics of the participants and measure alcohol and tobacco use and behavioural variables (Alcohol Use Disorders Identification Test (AUDIT) and the WHO STEPwise). The questionnaire was paper-based and translated into IsiXhosa (a local language predominantly used), allowing participants to be interviewed in their preferred language.

Data were collected face-to-face in private areas at local health facilities to ensure participants’ convenience. Interviews took place between August and September 2023, each lasting 15–20 min. The study achieved a 95% response rate. Appointments with participants were scheduled in line with their treatment collection dates, while others preferred that the interview be conducted in their preferred space. Participants did not incur any costs for participating in the study.

### 2.7. Data Management

The captured data were (i) entered into the Statistical Package for Social Sciences (SPSS) software version 30 (SPSS Inc., Chicago, IL, USA); (ii) cleaned to address errors, inconsistencies and missing values; (iii) coded by assigning numerical codes to categorical variables; (iv) validated to ensure data accuracy and consistency; and (v) stored securely, ensuring confidentiality and integrity.

### 2.8. Data Analysis

Data were captured and analysed using IBM SPSS Statistics version 30 (IBM Corp., Armonk, NY, USA). Categorical variables were summarised using frequencies (*n*) and percentages (%), and the results were presented in tables and figures.

Alcohol use was classified according to the World Health Organization Alcohol Use Disorders Identification Test (AUDIT) guidelines as low-risk drinking (AUDIT score 0–7), hazardous drinking (8–15), harmful drinking (16–19), and probable alcohol dependence (AUDIT score ≥ 20) [17]. The AUDIT total score was calculated using the standard WHO scoring algorithm by summing responses to all ten AUDIT items. Alcohol consumption status (never vs. any alcohol use) was derived from AUDIT Question 1 (“How often do you have a drink containing alcohol?”), whereas AUDIT risk categories were derived from the total AUDIT score. Consequently, the descriptive analysis of alcohol consumption status and the analysis of AUDIT risk categories were based on different outcome variables and represent different measures of alcohol use and were analysed separately throughout the study.

Associations between sociodemographic characteristics and the four AUDIT risk categories, as well as smoking status, were initially assessed using cross-tabulations and Pearson’s chi-square tests. Where expected cell counts were sparse, the Fisher–Freeman–Halton exact test with Monte Carlo estimation (100,000 samples; 95% confidence level) was used.

Ordinal logistic regression was initially performed using the four AUDIT risk categories. However, the proportional odds assumption was violated (Test of Parallel Lines, *p* < 0.001), indicating that the ordinal model was inappropriate. Although multinomial logistic regression was considered, the primary objective of the study was to identify factors associated with clinically significant alcohol use rather than to distinguish between individual AUDIT severity categories. Therefore, alcohol use was dichotomised using the established AUDIT cut-off (AUDIT score < 8 vs. ≥8), classifying participants as low-risk drinkers and risky drinkers. This approach is consistent with the validated interpretation of the AUDIT and enabled the use of binary logistic regression to estimate crude and adjusted odds ratios with 95% confidence intervals.

A sensitivity analysis was performed after excluding participants who reported never consuming alcohol on the AUDIT frequency question (Question 1) but had a total AUDIT score of 8 or greater, indicating internally inconsistent responses. The primary regression analyses were repeated to evaluate the robustness of the findings.

### 2.9. Ethical and Legal Considerations

The study adhered to the provisions of the Helsinki Declaration. Ethical approval was obtained with references 093/2022 and NICR 2023 10498 from accredited committees. Gatekeeping was held by the Eastern Cape Department of Health. All participants provided informed consent before enrolling in the study.

### 2.10. Validity and Reliability

The Alcohol Use Disorder Identification Test (AUDIT) [17] and the adapted WHO-StepWise tool [18] were used. Internal consistency reliability was evaluated through Cronbach’s alpha, yielding a coefficient of 0.77. This evaluation was conducted to confirm that all items in the AUDIT tool consistently measure harmful drinking, probable alcohol dependence, and hazardous drinking. Additionally, a pilot study with 10 participants was conducted to assess the questionnaire’s feasibility, specifically regarding completion time and participant comprehension, thereby ensuring it accurately measured the intended outcomes.

## 3. Results

### 3.1. Socio-Demographic Characteristics

Table 1 summarises the socio-demographic characteristics of the study participants. The sample included slightly more females than males, with females making up 57% of the group. Age was collected in categorical form using predefined groups (18–26, 27–35, 36–44, 45–53, and >54 years). The largest age group was 45–53 years, accounting for 28.3% of participants, while the smallest was the 18–26 age group, representing 11.1%. Most participants had completed secondary school (36.1%), and a smaller portion (13.1%) had no formal education. The majority were single (36.5%), with only 8.2% being widowed. Christianity was the most common religion (50.8%), followed by Islam (30.3%), African religions (15.6%), and other religions (3.3%).

Employment was common, with 61.5% of participants working. Nearly half of the participants (46.3%) resided in rural areas, and a smaller proportion (13.1%) lived in informal settlements. The majority fell into the low-income category (66.8%), with just a small fraction (3.7%) earning a high income.

### 3.2. Alcohol Consumption

Table 2 summarises alcohol consumption status according to gender. Similar proportions of males (56.2%) and females (56.1%) reported consuming alcohol, whereas 43.8% of males and 43.9% of females reported never consuming alcohol. There was no statistically significant association between gender and alcohol consumption status (chi-square test, *p* = 0.842), indicating that alcohol consumption did not differ by gender in this study population.

**Figure 1:**
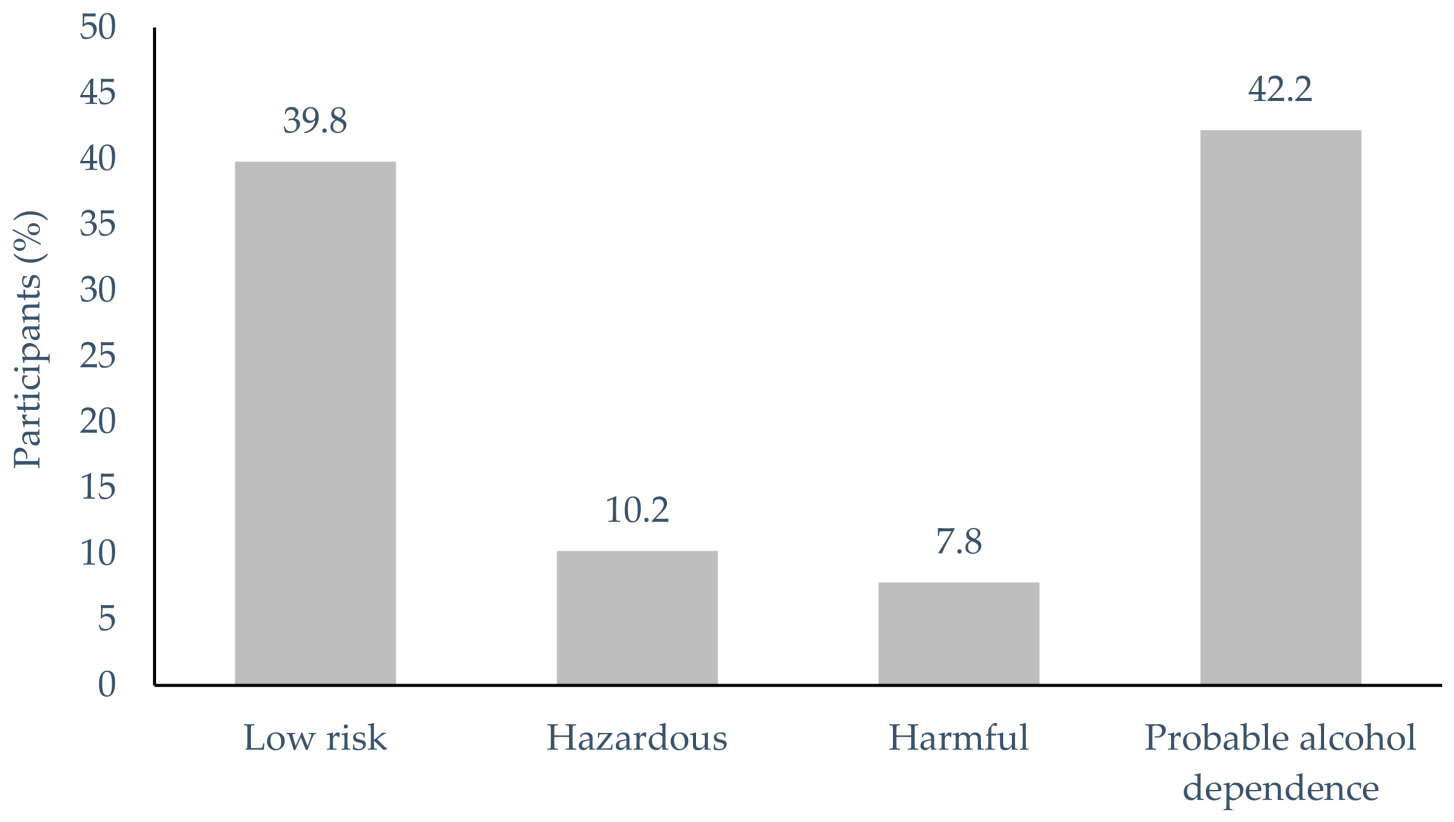
Distribution of participants according to WHO AUDIT alcohol risk categories.

Figure 1 illustrates the distribution of participants across the four WHO AUDIT alcohol risk categories. Overall, 39.8% of participants were classified as low-risk drinkers, 10.2% as hazardous drinkers, 7.8% as harmful drinkers, and 42.2% as having probable alcohol dependence. The largest proportion of participants were classified as having probable alcohol dependence (42.2%), followed closely by those in the low-risk drinking category (39.8%). Hazardous and harmful drinking accounted for a further 18.0% of the study population. These findings indicate a substantial burden of high-risk alcohol use within the study population and justify further investigation of the sociodemographic factors associated with risky drinking.

Table 3 presents the distribution of participants across the four AUDIT risk categories, by selected sociodemographic characteristics. Among the age groups, participants aged 45–53 years had the highest proportion in the low-risk drinking category (43.5%), whereas those aged 18–26 years had the highest proportion in the hazardous drinking category (18.5%). The highest proportions of harmful drinking and probable alcohol dependence were observed among participants aged 54 years and older (9.8% and 39.0%, respectively) and those aged 36–44 years (8.2% and 47.5%, respectively). However, there was no statistically significant association between age group and AUDIT risk category (Fisher–Freeman–Halton exact test, *p* = 0.906).

A statistically significant association was observed between gender and AUDIT risk category (χ^2^ = 11.65, *p* = 0.009). Compared with females, males had higher proportions of hazardous drinking (15.2% vs. 6.5%) and harmful drinking (12.4% vs. 4.3%), whereas females had higher proportions of low-risk drinking (42.4% vs. 36.2%) and probable alcohol dependence (46.8% vs. 36.2%).

Participants with secondary education had the highest proportion in the low-risk drinking category (47.7%), whereas those with no formal education had the highest proportion of probable alcohol dependence (50.0%). Participants with primary and tertiary education showed identical distributions across the AUDIT categories in this sample. Verification of the source dataset confirmed that these values reflected the observed data rather than a transcription error. Educational level was not significantly associated with AUDIT risk category (Fisher–Freeman–Halton exact test, *p* = 0.847).

With respect to marital status, single participants had the highest proportion of low-risk drinking (50.6%), whereas separated participants had the highest proportion of probable alcohol dependence (56.4%). Married participants showed the highest proportion of harmful drinking (10.7%). However, no statistically significant association was observed between marital status and AUDIT risk category (Fisher–Freeman–Halton exact test, *p* = 0.149).

Employment status was significantly associated with AUDIT risk category (χ^2^ = 9.23, *p* = 0.026). Employed participants had a higher proportion of probable alcohol dependence than unemployed participants (48.0% vs. 33.0%) and also a higher proportion of harmful drinking (9.3% vs. 5.3%). In contrast, unemployed participants had a higher proportion of low-risk drinking (51.1% vs. 32.7%).

No statistically significant associations were observed for age group (*p* = 0.906), educational level (*p* = 0.847), or marital status (*p* = 0.149). Variables examined in the bivariate analyses were subsequently entered into a multivariable logistic regression model to determine their independent associations with risky alcohol consumption.

A multivariable binary logistic regression analysis was performed to identify sociodemographic factors independently associated with risky alcohol consumption (Table 4). The overall model was statistically significant (χ^2^ = 28.69, df = 13, *p* = 0.007), indicating that the included variables significantly improved the prediction of risky alcohol consumption. The model demonstrated acceptable goodness-of-fit according to the Hosmer–Lemeshow test (χ^2^ = 9.99, df = 8, *p* = 0.265), with a Nagelkerke R^2^ of 0.150.

In the univariable analysis, employment status (*p* = 0.005) and marital status (*p* = 0.028) were significantly associated with risky alcohol consumption, whereas gender, age group, and educational level were not.

After adjusting for all covariates, employment status and marital status remained independently associated with risky alcohol consumption. Employed participants had approximately twice the odds of risky alcohol consumption compared with unemployed participants (AOR = 2.03, 95% CI: 1.11–3.73; *p* = 0.022).

Compared with single participants, married individuals had 2.61 times higher odds of risky alcohol consumption (AOR = 2.61, 95% CI: 1.25–5.45; *p* = 0.011). Separated participants had the highest likelihood of risky alcohol consumption, with more than fivefold increased odds (AOR = 5.19, 95% CI: 1.98–13.60; *p* = 0.001), while divorced participants had over three times higher odds (AOR = 3.32, 95% CI: 1.05–10.49; *p* = 0.041). However, these estimates should be interpreted cautiously because they were derived from relatively small groups and were accompanied by wide confidence intervals. No significant difference was observed between widowed and single participants (AOR = 1.45, 95% CI: 0.48–4.37; *p* = 0.507).

Gender, age group, and educational level were not independently associated with risky alcohol consumption after adjustment for potential confounders (*p* > 0.05 for all comparisons), although participants aged 45–53 years showed a non-significant trend towards lower odds of risky alcohol consumption than those aged 18–26 years (AOR = 0.34, 95% CI: 0.11–1.06; *p* = 0.063).

The multivariable model explained approximately 15.0% of the variation in risky alcohol consumption (Nagelkerke R^2^ = 0.150) and correctly classified 69.3% of participants.

A sensitivity analysis was performed after excluding 44 participants who reported never consuming alcohol on AUDIT Question 1 but had a total AUDIT score ≥ 8, indicating internally inconsistent AUDIT responses. After exclusion, the prevalence of risky drinking decreased from 60.2% to 51.5%. However, the multivariable findings remained materially unchanged. Employment status remained independently associated with risky drinking (AOR = 2.08, 95% CI 1.17–3.69; *p* = 0.013), and marital status also remained significantly associated with risky drinking (overall *p* = 0.028), supporting the robustness of the primary findings.

### 3.3. Tobacco Use

Table 5 presents the distribution of smoking status by participants’ sociodemographic characteristics, expressed as row percentages. Smoking prevalence varied significantly across age groups (*p* = 0.041). The highest smoking prevalence was observed among participants aged 18–26 years (55.6%), followed by those aged 36–44 years (52.5%), whereas participants aged 45–53 years had the lowest prevalence (29.0%).

There was no statistically significant association between gender and smoking status (*p* = 0.239). Although 48.6% of males were smokers compared with 41.0% of females, this difference was not statistically significant.

Educational level was significantly associated with smoking status (*p* = 0.006). Participants with primary (54.8%) and tertiary (54.8%) education had the highest smoking prevalence, whereas those with secondary school education had the lowest smoking prevalence (30.7%). No consistent linear trend in smoking prevalence across educational levels was observed.

Employment status was also significantly associated with smoking status (*p* < 0.001). More than half of employed participants were smokers (55.3%), compared with only 26.6% of unemployed participants. Conversely, the proportion of non-smokers was substantially higher among unemployed participants (73.4%) than among employed participants (44.7%).

Table 6 presents the univariable and multivariable logistic regression analyses of socio-demographic factors associated with current tobacco smoking. In the univariable analyses, age group (*p* = 0.046), educational level (*p* = 0.007), and employment status (*p* < 0.001) were significantly associated with current smoking, whereas gender was not (*p* = 0.239).

In the multivariable logistic regression model, only employment status remained independently associated with current tobacco smoking after adjusting for gender, age group, and educational level. Employed participants had significantly higher odds of being current smokers than unemployed participants (AOR = 3.75, 95% CI: 1.98–7.08, *p* < 0.001).

Although educational level remained significant overall (overall *p* = 0.016), none of the individual educational categories differed significantly from the reference category after adjustment. Similarly, neither age group (overall *p* = 0.149) nor gender (AOR = 1.69, 95% CI: 0.94–3.03, *p* = 0.081) was independently associated with current smoking.

The final multivariable logistic regression model was statistically significant (χ^2^ = 40.77, df = 9, *p* < 0.001), demonstrated good calibration (Hosmer-Lemeshow *p* = 0.616), and correctly classified 70.1% of participants.

## 4. Discussion

This study included a slightly higher proportion of female participants (57.0%) than males (43.0%), which is comparable to the national population distribution reported by Statistics South Africa, where females constitute approximately 52% of the population [16]. Christianity was the predominant religion among participants (50.8%), followed by Islam (30.3%), African religions (15.6%), and other religions (3.3%). Compared with national estimates, the proportion of Islamic participants in this study was higher than that reported for the South African population (16). The religion variable was verified against the source dataset, and no coding or transcription errors were identified. This difference, therefore, likely reflects the demographic characteristics of the study setting and sampling strategy rather than the national religious distribution.

### 4.1. Alcohol Use

This study examined the sociodemographic patterns of alcohol and tobacco use among PLWH who had achieved viral load suppression in the Eastern Cape in South Africa. This study used the AUDIT screening tool to classify participants according to alcohol use risk and revealed that the prevalence of alcohol consumption among PLWH who achieved viral load suppression was 56.1%.

The relatively high proportion of participants classified as having probable alcohol dependence is concerning, particularly because all participants had achieved viral load suppression. This finding suggests that successful HIV treatment does not necessarily eliminate behaviours that may compromise long-term health outcomes. Similar studies conducted among people living with HIV in sub-Saharan Africa have reported a high burden of hazardous alcohol use, although prevalence estimates vary considerably depending on the study population, screening instrument, and setting [18]. The persistence of harmful alcohol use among people receiving antiretroviral therapy may reflect broader social and structural determinants, including socioeconomic hardship, unemployment, psychological distress, alcohol availability, social drinking norms, and limited access to behavioural interventions. In many resource-limited settings, routine HIV services primarily focus on antiretroviral adherence and viral suppression, whereas systematic screening and management of alcohol use disorders remain limited [19]. These findings highlight the importance of integrating routine alcohol screening, brief behavioural interventions, and referral pathways into comprehensive HIV care.

For the regression analyses, participants were subsequently classified as low-risk drinkers (AUDIT score < 8) and risky drinkers (AUDIT score ≥ 8). Probable alcohol dependence, as identified by the AUDIT screening tool, represents individuals who require further clinical assessment for alcohol use disorder rather than a definitive clinical diagnosis [20]. In 2016, a quarter of a billion globally, or one in every twenty persons aged 15 to 64, used a substance at least once [21]. Kaswa and de Villiars further highlight that the global prevalence of substance use is estimated to be 5.2%, which has been consistent since 2011 [22].

Excessive alcohol consumption is a predisposing factor for participation in a range of high-risk activities, such as hazardous sexual activity, which might promote transmission and could affect the response to antiretroviral treatment [23]. Alcohol use disorders have immediate and long-term effects on the physical, mental, and socioeconomic elements of an individual’s life [24], and PLWH are significantly affected. Among people living with HIV, continued hazardous alcohol use has also been associated with poorer retention in care, reduced medication adherence, increased sexual risk behaviours, and a higher burden of non-communicable diseases despite virological suppression. For example, substance use compromises the immune system, predisposes persons to bacterial diseases like TB, and may cause liver damage and disrupt the metabolism of antiviral medications essential for viral load suppression [25]. Maintaining long-term viral suppression is vital for persons with HIV to achieve optimal individual health outcomes and avoid transmission [26].

Although females constituted a larger proportion of the study sample, males exhibited higher proportions of hazardous drinking (15.2% vs. 6.5%) and harmful drinking (12.4% vs. 4.3%) than females in the bivariate analysis. The findings of the present study align closely with those of a prior investigation conducted in Europe, which revealed a significantly higher prevalence of hazardous drinking among men compared to women. Specifically, the study reported rates of 30.2% (95% CI: 29.1–31.4%) for men and 18.6% (95% CI: 17.7–19.4%) for women, highlighting the gender disparity in drinking behaviours within this population [27]. However, after adjustment for other sociodemographic factors in this study, gender was not independently associated with risky alcohol consumption, suggesting that the apparent differences observed in the unadjusted analysis were explained by other participant characteristics.

In addition to gender differences, this study found that employment status was significantly associated with alcohol risk in both the bivariate and multivariable analyses. Employed participants had higher odds of risky alcohol consumption than unemployed participants after adjustment for other sociodemographic factors, suggesting that employment status may independently influence alcohol consumption patterns among people living with HIV who have achieved viral suppression. This finding may reflect differences in disposable income, workplace social environments, or other socioeconomic factors that warrant further investigation. However, because of the cross-sectional design, these findings should be interpreted as associations rather than evidence of causal relationships. This connection emphasises how work status may influence alcohol consumption behaviours, since employed persons may have distinct social and economic constraints from their unemployed counterparts [28].

The relationships between HIV, substance use and abuse, and employment are complex and diverse. Each of these variables affects the others, often worsening health inequities and affecting people’s overall quality of life. Research shows that steady employment is positively connected with improved health outcomes, including greater rates of HIV testing, linkage to care, retention in treatment, and medication adherence [29]. However, PLWH struggling with substance use face additional challenges, including greater difficulty maintaining employment due to ill health and workplace stigma. Employment status may alter the patterns of alcohol intake and reliance on PLWH, owing to social drinking that enhances their risk levels [30]. Marital status was also independently associated with risky alcohol consumption in the multivariable analysis. Although no statistically significant association was observed in the descriptive analysis of the four AUDIT risk categories, adjustment for potential confounding variables identified marital status as an independent predictor. This finding illustrates the importance of multivariable modelling in identifying associations that may not be apparent in unadjusted analyses.

A sensitivity analysis excluding participants with internally inconsistent AUDIT responses reduced the estimated prevalence of risky drinking from 60.2% to 51.5%. However, the independent associations between employment status, marital status, and risky drinking remained materially unchanged. These findings provide additional reassurance that the principal conclusions of the study are robust despite the presence of a small number of inconsistent AUDIT responses.

### 4.2. Smoking

The link between HIV, age, and smoking is complicated and multidimensional, with important implications for the health of people living with HIV. This study revealed variations in smoking prevalence across age groups but did not demonstrate a consistent increase in smoking with advancing age. These findings are broadly consistent with a South African study conducted between 2012 and 2013, which reported higher smoking prevalence among older people living with HIV [31]. However, direct comparisons should be interpreted cautiously because differences in study design, participant characteristics, geographical setting, and definitions of smoking status may account for some of the observed variation.

The present study found that 48.6% of male participants and 41.0% of female participants were current smokers. Although these proportions differ from those reported by Uthman et al. among people living with HIV in sub-Saharan Africa [32], direct comparisons should be interpreted with caution because the studies were conducted in different populations, settings, and time periods using different sampling strategies. Consequently, the present findings should not be interpreted as evidence of temporal changes in smoking prevalence but rather as reflecting the smoking patterns observed among virally suppressed people living with HIV in the current study population. Furthermore, after adjustment for potential confounders, gender was not independently associated with smoking status, indicating that the observed differences between males and females were not statistically significant.

Educational attainment has an inverse relationship with smoking prevalence among PLWH. Individuals who completed at least Grade 12 have a lower risk of being current smokers or using other tobacco products [8]. Findings from the current study align with these results, demonstrating that educational level is significantly associated with smoking behaviour (*p* = 0.006). Specifically, individuals with elementary and tertiary education were more likely to smoke, whereas those with secondary school education were more likely to be non-smokers.

The current study demonstrates a statistically significant relationship between employment status and tobacco usage (*p* = 0.001). Among employed participants in this current study, 55.3% were current smokers and 44.7% were not current smokers, compared with 26.6% current smokers and 73.4% not current smokers among unemployed participants. This study is consistent with the study conducted in Vietnam in 2013, which found statistical significance in smoking status by employment, age, gender, and HIV (*p* = 0.05) [33].

## 5. Conclusions

This study demonstrated that alcohol use and tobacco smoking remain common among virally suppressed people living with HIV receiving care in primary healthcare facilities in the Eastern Cape Province, South Africa. Although viral load suppression is an important indicator of successful HIV treatment, it does not fully reflect broader health behaviours that may influence long-term health outcomes. Based on the total AUDIT score, 60.2% of participants were classified as risky drinkers, and 44.3% were current smokers, highlighting the continued burden of substance use in this population.

After adjustment for potential confounding factors, employment status and marital status were independently associated with risky alcohol consumption, while employment status was independently associated with current smoking. These findings suggest that socioeconomic factors continue to influence substance use behaviours even among individuals who have achieved viral load suppression.

Routine screening for alcohol and tobacco use should therefore be integrated into comprehensive HIV care, together with brief behavioural interventions, counselling, smoking cessation support, and harm reduction strategies. Addressing substance use within HIV services may contribute to improved treatment adherence, better overall health, and enhanced quality of life among people living with HIV.

## 6. Strengths and Limitations of the Study

This study has several strengths. It included participants from 11 primary healthcare facilities in the Eastern Cape Province, providing a broad representation of people living with HIV who had achieved viral load suppression in the study setting. Alcohol use was assessed using the validated Alcohol Use Disorders Identification Test (AUDIT), and the study examined both alcohol and tobacco use together with their sociodemographic correlates. Furthermore, multivariable logistic regression analyses were performed to adjust for potential confounding factors and identify independent predictors of risky alcohol use and current smoking.

The findings, however, should be interpreted in light of several limitations. First, the cross-sectional design precludes establishing causal relationships between sociodemographic characteristics and substance use. Second, alcohol and tobacco use were measured using self-reported information and may therefore be subject to recall bias and social desirability bias, potentially resulting in underreporting or misclassification of substance use. In addition, although the AUDIT is a validated screening instrument, some participants provided internally inconsistent responses across individual AUDIT items (e.g., reporting never consuming alcohol on the frequency question while indicating alcohol-related behaviours on subsequent items). The primary analyses retained these internally inconsistent responses to preserve the standard WHO AUDIT scoring algorithm. However, a sensitivity analysis excluding these participants produced materially similar regression results, indicating that although the estimated prevalence of risky drinking was affected, the principal associations identified in the study remained robust. The inconsistencies may reflect recall bias, misunderstanding of individual questions, or social desirability bias. Furthermore, the AUDIT identifies probable alcohol dependence rather than clinically confirmed alcohol dependence.

The study was conducted exclusively among virally suppressed people living with HIV, which may limit the generalisability of the findings to all people living with HIV. Finally, although participants were recruited from 11 primary healthcare facilities, the anonymised dataset did not include a facility identifier, precluding adjustment for clustering at the facility level. Consequently, residual within-facility correlation cannot be excluded and should be considered when interpreting the regression estimates.

## Figures and Tables

**Table 1:** Socio-demographics of participants involved in the study.

Sociodemographic Variables	*n*	%	95% CI
Gender			
Male	105	43.0	36.8–49.2
Female	139	57.0	50.8–63.2
Age group (years)			
18–26	27	11.1	7.1–15.1
27–35	46	18.9	14.0–23.8
36–44	61	25.0	19.6–30.4
45–53	69	28.3	22.6–34.0
≥54	41	16.8	12.1–21.5
Educational level			
None	32	13.1	8.9–17.3
Primary	62	25.4	19.9–30.9
Secondary school	88	36.1	30.0–42.2
Tertiary	62	25.4	19.9–30.9
Marital status			
Single	89	36.5	30.4–42.6
Married	75	30.7	25.0–36.4
Separated	39	16.0	11.4–20.6
Divorced	21	8.6	5.1–12.1
Widowed	20	8.2	4.8–11.6
Employment status			
Yes	150	61.5	55.4–67.6
No	94	38.5	32.4–44.6
Location			
Rural	113	46.3	40.1–52.5
Urban	99	40.6	34.5–46.7
Informal settlement	32	13.1	8.9–17.3
Income			
Low (R0–R5000/month)	163	66.8	60.9–72.7
Moderate (R5001–R15,000/month)	72	29.5	23.8–35.2
High (≥R15,000/month)	9	3.7	1.3–6.1
Religion			
Christianity	124	50.8	44.5–57.1
Islam	74	30.3	24.6–36.0
African Religions	38	15.6	11.1–20.1
Other	8	3.3	1.1–5.5

**Table 2:** Distribution of alcohol consumption status (never vs. any alcohol use) according to gender.

Gender	Never Consumed Alcohol *n* (%)	Consumes Alcohol *n* (%)	Total
Male	46 (43.8)	59 (56.2)	105
Female	61 (43.9)	78 (56.1)	139
**Total**	107 (43.9)	137 (56.1)	244

**Table 3:** Distribution of AUDIT risk categories according to sociodemographic characteristics.

Predictor	Low-Risk *n* (%)	Hazardous Drinking *n* (%)	Harmful Drinking *n* (%)	Probable Alcohol Dependence *n* (%)	*p*-Value
Gender					0.009
Female	59 (42.4)	9 (6.5)	6 (4.3)	65 (46.8)	
Male	38 (36.2)	16 (15.2)	13 (12.4)	38 (36.2)	
Age group					0.906 *
18–26	9 (33.3)	5 (18.5)	1 (3.7)	12 (44.4)	
27–35	18 (39.1)	4 (8.7)	4 (8.7)	20 (43.5)	
36–44	24 (39.3)	3 (4.9)	5 (8.2)	29 (47.5)	
45–53	30 (43.5)	8 (11.6)	5 (7.2)	26 (37.7)	
≥54	16 (39.0)	5 (12.2)	4 (9.8)	16 (39.0)	
Educational level					0.847 *
No formal education	11 (34.4)	2 (6.3)	3 (9.4)	16 (50.0)	
Primary	22 (35.5)	7 (11.3)	5 (8.1)	28 (45.2)	
Secondary	42 (47.7)	9 (10.2)	6 (6.8)	31 (35.2)	
Tertiary	22 (35.5)	7 (11.3)	5 (8.1)	28 (45.2)	
Employment status					0.026
Unemployed	48 (51.1)	10 (10.6)	5 (5.3)	31 (33.0)	
Employed	49 (32.7)	15 (10.0)	14 (9.3)	72 (48.0)	
Marital status					0.149 *
Single	45 (50.6)	10 (11.2)	4 (4.5)	30 (33.7)	
Married	27 (36.0)	5 (6.7)	8 (10.7)	35 (46.7)	
Separated	9 (23.1)	5 (12.8)	3 (7.7)	22 (56.4)	
Divorced	6 (28.6)	3 (14.3)	2 (9.5)	10 (47.6)	
Widowed	10 (50.0)	2 (10.0)	2 (10.0)	6 (30.0)	

Footnote: * *p*-values were obtained using the Fisher–Freeman–Halton exact test with Monte Carlo estimation based on 100,000 sampled tables (95% confidence interval).

**Table 4:** Univariable and multivariable logistic regression analysis of factors associated with risky alcohol consumption.

Predictor	Crude OR (95% CI)	*p*-Value	Adjusted OR (95% CI)	*p*-Value
Gender		0.323		0.723
Female (Ref.)	1.00	—	1.00	—
Male	1.30 (0.77–2.19)	0.323	1.11 (0.62–2.01)	0.723
Age group		0.927		0.425
18–26 years (Ref.)	1.00	—	1.00	—
27–35 years	1.28 (0.46–3.54)	0.634	0.57 (0.19–1.70)	0.312
36–44 years	1.00 (0.42–2.36)	0.992	0.42 (0.14–1.29)	0.130
45–53 years	0.99 (0.44–2.22)	0.974	0.34 (0.11–1.06)	0.063
≥54 years	0.83 (0.38–1.83)	0.647	0.50 (0.14–1.74)	0.274
Educational level		0.303		0.192
No formal education (Ref.)	1.00	—	1.00	—
Primary	1.05 (0.43–2.57)	0.915	1.44 (0.52–3.99)	0.488
Secondary school	0.60 (0.31–1.17)	0.137	0.60 (0.28–1.28)	0.183
Tertiary	1.00 (0.48–2.09)	1.000	1.05 (0.45–2.46)	0.903
Employment status		0.005		0.022
Unemployed (Ref.)	1.00	—	1.00	—
Employed	2.15 (1.27–3.65)	0.005	2.03 (1.11–3.73)	0.022
Marital status		0.028		0.008
Single (Ref.)	1.00	—	1.00	—
Married	1.82 (0.97–3.41)	0.062	2.61 (1.25–5.45)	0.011
Separated	3.41 (1.45–8.00)	0.005	5.19 (1.98–13.60)	0.001
Divorced	2.56 (0.91–7.19)	0.075	3.32 (1.05–10.49)	0.041
Widowed	1.02 (0.39–2.70)	0.964	1.45 (0.48–4.37)	0.507

Footnote: OR, odds ratio; AOR, adjusted odds ratio; CI, confidence interval; Ref., reference category.

**Table 5:** Association between sociodemographic characteristics and current smoking status.

Variables	Non-Smoker *n* (%)	Smoker *n* (%)	Total	*p*-Value
Age group				0.041
18–26 years	12 (44.4)	15 (55.6)	27	
27–35 years	25 (54.3)	21 (45.7)	46	
36–44 years	29 (47.5)	32 (52.5)	61	
45–53 years	49 (71.0)	20 (29.0)	69	
≥54 years	21 (51.2)	20 (48.8)	41	
Total	136 (55.7)	108 (44.3)	244	
Gender				0.239
Female	82 (59.0)	57 (41.0)	139	
Male	54 (51.4)	51 (48.6)	105	
Total	136 (55.7)	108 (44.3)	244	
Educational level				0.006
No formal education	19 (59.4)	13 (40.6)	32	
Primary	28 (45.2)	34 (54.8)	62	
Secondary school	61 (69.3)	27 (30.7)	88	
Tertiary	28 (45.2)	34 (54.8)	62	
Total	136 (55.7)	108 (44.3)	244	
Employment status				<0.001
Employed	67 (44.7)	83 (55.3)	150	
Unemployed	69 (73.4)	25 (26.6)	94	
Total	136 (55.7)	108 (44.3)	244	

**Table 6:** Univariable and multivariable logistic regression analysis of socio-demographic factors associated with current tobacco smoking among virally suppressed people living with HIV.

Predictor	Crude OR (95% CI)	*p*-Value	Adjusted OR (95% CI)	*p*-Value
Gender		0.239		0.081
Female (Ref.)	1.00	—	1.00	—
Male	1.36 (0.82–2.26)	0.239	1.69 (0.94–3.03)	0.081
Age group		0.046		0.149
18–26 years (Ref.)	1.00	—	1.00	—
27–35 years	0.67 (0.26–1.75)	0.415	0.82 (0.29–2.28)	0.698
36–44 years	0.88 (0.36–2.19)	0.788	0.98 (0.36–2.64)	0.960
45–53 years	0.33 (0.13–0.82)	0.017	0.43 (0.16–1.18)	0.100
≥54 years	0.76 (0.29–2.02)	0.585	1.15 (0.38–3.51)	0.802
Educational level		0.007		0.016
No formal education (Ref.)	1.00	—	1.00	—
Primary	1.78 (0.75–4.21)	0.194	1.63 (0.62–4.27)	0.324
Secondary school	0.65 (0.28–1.50)	0.309	0.49 (0.19–1.27)	0.143
Tertiary	1.78 (0.75–4.21)	0.194	0.92 (0.34–2.53)	0.873
Employment status		<0.001		<0.001
Unemployed (Ref.)	1.00	—	1.00	—
Employed	3.42 (1.95–5.98)	<0.001	3.75 (1.98–7.08)	<0.001

Footnote: OR, odds ratio; AOR, adjusted odds ratio; CI, confidence interval; Ref., reference category.

## Data Availability

All data analysed in this study will be made available upon request from the corresponding author.

## Abbreviations

The following abbreviations are used in this manuscript:

HIV	Human Immunodeficiency Virus
PLWH	People Living with Human Immunodeficiency Virus
ART	Antiretroviral Treatment
VL	Viral Load
AUDIT	Alcohol Use Disorders Identification Test
